# Engineered Biosensors for Diagnosing Multidrug Resistance in Microbial and Malignant Cells

**DOI:** 10.3390/bios13020235

**Published:** 2023-02-07

**Authors:** Niharika G. Jha, Daphika S. Dkhar, Sumit K. Singh, Shweta J. Malode, Nagaraj P. Shetti, Pranjal Chandra

**Affiliations:** 1 School of Biochemical Engineering, Indian Institute of Technology (BHU) Varanasi, Varanasi 221005, Uttar Pradesh, India; 2 Center for Energy and Environment, School of Advanced Sciences, KLE Technological University, Hubballi 580031, Karnataka, India; 3 University Center for Research & Development (UCRD), Chandigarh University, Mohali 140413, Panjab, India

**Keywords:** multidrug resistance, MDR, biosensors, sensors, drugs, drug efflux pump, antibiotics, antimicrobial, cancer, microorganisms

## Abstract

To curtail pathogens or tumors, antimicrobial or antineoplastic drugs have been developed. These drugs target microbial/cancer growth and survival, thereby improving the host’s health. In attempts to evade the detrimental effects of such drugs, these cells have evolved several mechanisms over time. Some variants of the cells have developed resistances against multiple drugs or antimicrobial agents. Such microorganisms or cancer cells are said to exhibit multidrug resistance (MDR). The drug resistance status of a cell can be determined by analyzing several genotypic and phenotypic changes, which are brought about by significant physiological and biochemical alterations. Owing to their resilient nature, treatment and management of MDR cases in clinics is arduous and requires a meticulous approach. Currently, techniques such as plating and culturing, biopsy, gene sequencing, and magnetic resonance imaging are prevalent in clinical practices for determining drug resistance status. However, the major drawbacks of using these methods lie in their time-consuming nature and the problem of translating them into point-of-care or mass-detection tools. To overcome the shortcomings of conventional techniques, biosensors with a low detection limit have been engineered to provide quick and reliable results conveniently. These devices are highly versatile in terms of analyte range and quantities that can be detected to report drug resistance in a given sample. A brief introduction to MDR, along with a detailed insight into recent biosensor design trends and use for identifying multidrug-resistant microorganisms and tumors, is presented in this review.

## 1. Introduction

The introduction should briefly place the study in a broad context and highlight its importance. Multidrug resistance (MDR) is a globally recognized pressing health concern that has rendered several drugs ineffective against many hitherto treatable diseases. Examples include tuberculosis, malaria, urinary tract infection, gonorrhea, pneumonia, and fever, which have increasingly become more difficult to treat due to MDR [1]. The underlying reason for the emergence of the multi-drug resistant strains of microorganisms can be attributed in part to the presence of antimicrobial (AM)/antibiotic residues in the environment owing to indiscriminate use of AM/antibiotics in human and veterinary medicines, livestock farming, and to a certain extent on personal negligence of the patients in consuming antibiotics [2,3,4]. MDR is becoming a major cause of concern because relatively fewer new antibiotics are being added to the developmental pipelines of the pharmaceutical industry while the existing ones are becoming redundant. For instance, a recent study revealed that 45 antibiotic drugs are at various stages of the clinical development pipelines currently [5]. However, around 26% of *Escherichia coli* clinical isolates from the neonatal wards of hospitals exhibited MDR [6], suggesting a disparity in the rates of newer antibiotic discovery versus the emergence of MDR.

MDR has also been recognized as a stumbling block in the effective treatment of cancers. MDR in cancers is the primary reason for tumor recurrence and refractory cancers [7,8]. While a definite underlying theory for the development of MDR in cancers is still elusive, initially, MDR exhibited by cancer cells was approached by borrowing insights from microbial MDR [9]. As with microbial MDR, most drug-resistant cancerous cells can evade the effects of various prescribed anticancer agents. However, unlike microbial MDR, these cells can develop cross-resistance to drugs that may be structurally and functionally unrelated [8]. Nevertheless, with the rampant increase in the number and types of cancers, the effects of MDR inevitably impact the treatment regime and the quality of life of patients adversely, as it limits the efficacious and extended use of drugs [10].

**Figure 1:**
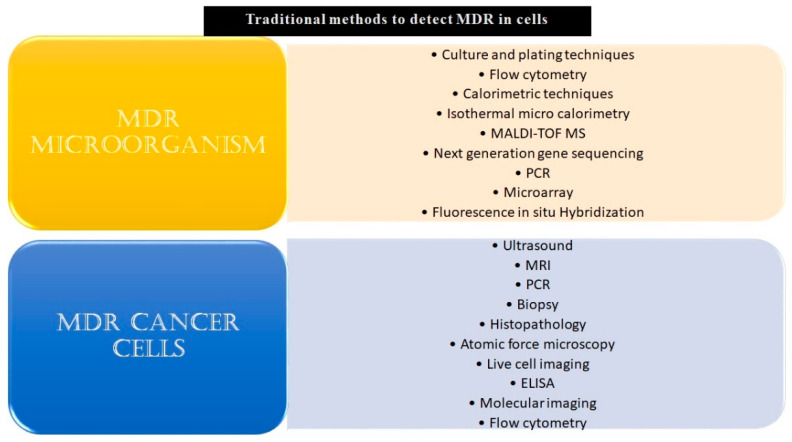
Conventional methods to detect MDR in microbial or malignant systems. The conventional and modern methods which have been used in the detection of MDR specimens have been listed above. Some of these techniques, for example, culturing and plating techniques and biopsy, are considered gold standards for confirmation of drug resistance in a given sample. Though these reliably yield results, a major drawback of most of these methods is that they are time-consuming and fail to give real-time data. Other drawbacks include the requirement of skilled labor, laboratory setup, cumbersome procedures, and an inability to carry out large-scale testing.

Given the challenges MDR poses by burdening healthcare systems, it is highly pertinent to develop technologies that can opportunistically screen/identify MDR strains, especially during the early stages of their onset, and monitor them. The existing methods to detect microbial and cancer MDR include culturing and plating techniques, ELIZA, biopsy, MRI, PCR, histopathology, next-generation sequencing techniques, and others, as listed in Figure 1.

A major shortcoming of these techniques is that they are time-intensive and hence cannot be used for real-time analysis and monitoring [11,12,13]. Other drawbacks include invasiveness, low sensitivities and selectivities, a high cost of sample processing, and the requirement of sophisticated lab infrastructure and skilled professionals to run the tests [14,15,16,17]. Similarly, most conventional techniques that are used in MDR cancer detection focus on phenotypic characteristics rather than detecting subtle but significant changes at the genetic and epigenetic levels, rendering them ineffective for the early detection of MDR [18,19,20,21].

Biosensors have emerged as an attractive option for yielding specific and selective diagnoses for MDR-related diseases [17]. Biosensor devices capitalize on the underlying physicochemical aspects of biological systems and are developed by integrating engineering, chemistry, and electronics concepts. The concept of biosensor devices is aptly reflected in the IUPAC’s definition of biosensors, which are described as devices that use specific biochemical reactions performed by biological entities like isolated enzymes, immune systems, tissues, organelles, or whole cells to detect chemical compounds, usually by electrical, thermal, or optical signals [22]. In essence, these devices identify the analyte of interest, that may be a part, or a product, of a biological system or reaction. With the help of a suitable signal processing methodology, biosensors convert the events of recognition of these moieties to measurable signal outputs that are further processed and amplified [23].

The current paradigm in biosensor development focuses on improving measurable attributes such as maximizing selectivity sensitivity, signal-to-noise ratio, reproducibility, and multiplexing and detecting an array of phenotypic traits/genotypic elements that contribute to the prevalence of MDR [15,24]. Biosensors are poised to serve as an excellent point of care diagnosis of MDR-related diseases [25,26].

In this review, we highlight various aspects of biosensor development that have helped in the detection/identification of MDR pathogens or cancer cells. Further, the concepts and the origins of MDR, and factors contributing to the emergence of these variants, are comprehensively discussed. Moreover, the application of biosensors to address the issue of microbial and cancer MDR is elaborately discussed. The present paper is expected to be of interest to those working in academia and industry on MDR mechanisms and the development of bioanalytical technologies for their detection. 

## 2. Multidrug Resistance in Microorganisms and Biosensors

### 2.1. MDR Glossary and Origin of Concept

Following the landmark serendipitous discovery of penicillin, many classes of AMs against many pathogenic organisms like bacteria, protozoa, fungi, helminths, and viruses were discovered [27]. Upon the acknowledgment of drug-resistant variants, terms were coined to describe the resistance of pathogens toward various AM agents. Though there are no hard and fast lines segregating these microbial groups, the Centre for Disease Control & Prevention (CDC) and the European Centre for Disease Control (ECDC) have put forward guidelines to define these terms [28]. 

As per the guidelines, antimicrobial categories were designed uniquely for the epidemiologically important microorganisms *Enterobacteriaceae, Staphylococcus aureus, Enterococcus spp., Acinetobacter, and Pseudomonas aeruginosa* considering the drugs classes that are pertinent to each microbe, and each AM category consists of one or more drug/AM agent [29]. For example, 17 categories were made for *S. aureus*, containing aminoglycosides, anti-MRSA (Methicillin Resistant *S. aureus*) cephalosporins, antistaphylococcal β-lactams, fluoroquinolones, macrolides, oxazolidinones, phenicols, phosphoric acid, streptogramins, folate pathway inhibitors, fucidanes, glycopeptides, glycylcyclines, lipopeptides, lincosamides, and tetracyclines [29].

**Figure 2:**
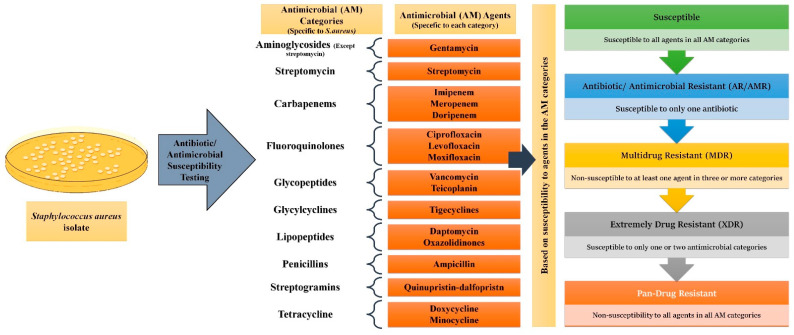
Classification of microorganisms based on drug resistance to antimicrobial (AM) agents: For all epidemiologically important microbes, AM categories of pertinence have been made. Each AM category contains one or more AM agents. For *Staphylococcus aureus* 17 categories have been made. Depending upon the response the particular microbe shows to a different agent in the different categories, they can be divided into AR/AMR, MDR, XDR, and PDR strains.

While antimicrobial resistance/antibiotic resistance (AMR/AR) is the ability of microorganisms to remain unaffected by a single AM or an antibiotic, MDR is the phenomenon where a microbe acquires non-susceptibility to at least one AM agent in a minimum of three categories [28]. XDR (Extremely Drug Resistant) pathogens remain susceptible to only one or two AM categories, whereas PDR (Pan Drug Resistant) expressing microbes show non-susceptibility to all agents in all AM categories [28]. Using *S. aureus* as an example, Figure 2 elaborates on how these distinctions are made.

### 2.2. Foundation of the Emergence of MDR 

The emergence of MDR in microbial species is attributed to AM/antibiotic resistance genes in their genome or plasmids of some microbes [30]. These genes have either been inherently present in the wild-type strain or may have developed by mutation. The lateral transfer of genes from some other microorganism or the host (i.e., from a producer organism) has also been proposed as a potential mechanism for acquiring such genes [30].

Exposure to AMs imposes a selection pressure on a heterogeneous population of microbes under which the survival of the fittest prevails [31]. This selection mechanism is also influenced by various parameters, such as the drug used on the microorganism (certain classes of drugs show more propensity at promoting AM resistance development than others), the dosage of the drug, the pharmacokinetic profile of the drug, the physiological state of the host, the fitness cost of resistance incurred by the pathogen, and the influence of non-resistant therapeutics [30]. The clustering of such genes in response to a series of selection pressures over time facilitates the emergence of MDR strains. Evidence suggests that MDR is preferred over AMR/AR because of the lower fitness cost [30,31,32]. 

The above discussion establishes that MDR is a natural phenomenon guided by evolutionary pressures. However, the central point of contention and concern is its rapid dissemination and increasing prevalence. This necessitates the development of appropriate tools for its rapid and accurate identification. 

### 2.3. Biochemical Basis of MDR in Microbes

The biochemical basis of microbial defenses against multiple drugs can be achieved by (a) alteration in the plasma membrane (PM) profile, (b) overcoming the drug action, and (c) alteration in the physiological state. It should be noted that these biochemical alterations may confer resistance to a single drug or more than one drug [33,34]

#### 2.3.1. Alteration in the PM Profile

For a microbe, the PM is a barrier serving as a selectively permeable interface to the external environment. This selective barrier profoundly impacts the exchange fluxes between the cell and its environment. Pathogens demonstrating MDR may have significantly altered the structure and composition of the PM. For instance, the additional mycolic acid present in the PM of XDR *Mycobacterium tuberculosis* renders it impervious to a plethora of drugs [35].These structural and compositional changes hinder the drugs from reaching their targets or achieving the optimal intracellular concentration.

Further, various transport proteins usually embedded in microbial species’ PM exhibit enhanced expression in MDR strains. Five major families of ABC transport proteins are associated with drug efflux through the PM of M/O. These families can be broadly divided into ATP-dependent and proton (or Na+) motive force-dependent transporters. While ABC (ATP Binding Cassette) transporters are ATP-dependent transport proteins, proton-dependent proteins include the SMR (Staphylococcal/ Small Multidrug Resistance), MF (Major Facilitator), RND (Resistance Nodulation Factor), and MATE (Multidrug and Toxic Compound Extrusion) family transporters. Any survival risk to the pathogens is averted by the active extrusion of drugs using the over-expressed families of transport proteins [35]. Apart from this mechanism, another strategy used by MDR species involves the down-regulation of porins, which are large transmembrane intrinsic proteins, restricting the passage of hydrophilic drugs like β-lactams and tetracyclines [2,31]. 

#### 2.3.2. Overcoming the Drug Action

If the drug overcomes the PM barrier and enters the cell, the following mechanisms are used to achieve resistance.

Target modification: The MDR species will bring about structural or configurational changes that result from genetic or epigenetic changes, thereby manifesting the following mechanisms leading to MDR. For instance: Changes in the penicillin-binding proteins by β-lactamase producing *S. aureus* strains conferred their resistance to penicillin, methicillin, and other drugs [31]; changes in the sequence of glycoproteinaceous pentapeptide conferred vancomycin resistance [30]. Expression of an alternate target for the drug, exhibiting a higher affinity that preferentially allows binding of the drug to the new target, or opting for target shunting by modification of the biochemical pathway, is also a method by which the cell can evade a drug’s action [31,36]

Drug modification: By this method, drugs may be rendered ineffective by resistant strains via inactivation of drugs by structural changes catalyzed by enzymes (e.g., enzymatic inactivation of antibiotics like kanamycin and tobramycin), or by post-translational modifications like phosphorylation, and adenylation and drug sequestration [30,37].

#### 2.3.3. Alteration in the Physiological State

The physiological state of pathogens also contributes to the emergence of MDR. A biofilm is a heterogeneous 3D structure embedded in a polymeric matrix comprising carbohydrates, nucleic acid, and proteins, commonly referred to as an extracellular polymeric matrix [38]. Biofilm formation has been associated with AM resilience [39]. This phenotype also promotes survival and persistence by providing a favorable niche protecting the microbes from external stresses like osmolarity, fluctuating pH, nutrient scarcity, mechanical forces, and even host immune cells [40,41]. MDR due to biofilm formation has been observed in pathogens like *E. coli, Pseudomonas aeruginosa, and Acinetobacter baumannii*. Biofilms encompass physiologically unique cells called persister cells [30]. These are metabolically inactive dormant cells tolerant to AM agents and do not undergo any genetic alteration to achieve such properties [42]. For example, the recalcitrance of chronic infections can be accredited to the presence of persister cells in biofilms. This observation is attributed to the survival of the persister cells, while most of the cells become non-viable due to antibiotics [42].

**Figure 3:**
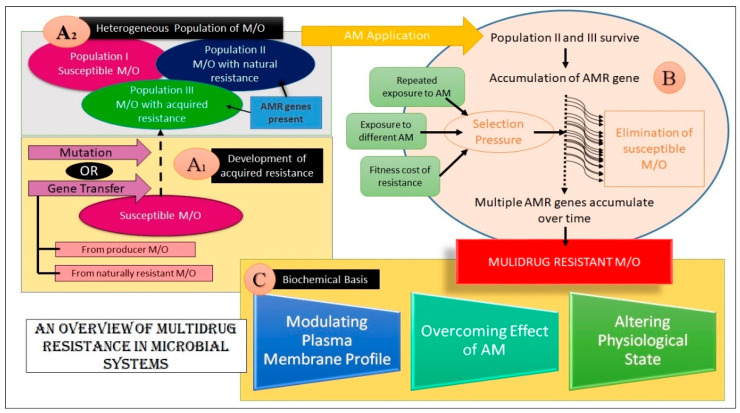
An overview of MDR in microorganisms. (Key: M/O- Microorganism(s), AM- Antimicrobial, AMR- Antimicrobial Resistance) The figure aims at explaining MDR emergence in microbes. Sections A1 and A2 depict how an assortment of microbes are present in the natural pool. This heterogeneous pool harbors some microbes that are susceptible to AM action, while some of them are resistant to AM agents. This resistance may be either innate or acquired. As shown, these AMR genes may be acquired from transfer from resistant/producer M/O or may be endowed by mutation. Block B shows how the resistant group survives upon AM application and procreates, leading to the accumulation of AMR genes. Over time, selection pressure is applied wherein the susceptible microbes get killed and multiple AMR genes accumulate in the M/O, imparting MDR. These genes dictate alterations to the subject by various means, finally yielding a multidrug-resistant microbe, as shown in section C.

An overview of the foundation and mechanisms of MDR development in microbial systems is summarized in Figure 3.

### 2.4. Use of Biosensors in Detecting MDR Microbes

The principle of biosensor design to identify MDR strains relies on detecting the genes/mutations conferring resistance. In addition, differential expression analysis of surrogate analytes that exhibit correlation with enhanced resistance or pathogenesis of the microbes is also monitored. Further, the MDR strains can be identified by analyzing altered physiological forms like biofilms. In this review, genotypic and phenotypic biosensors for detecting MDR microbial strains will be comprehensively discussed. 

#### 2.4.1. Genotypic Biosensor for MDR Microbes

The genome of an organism is the blueprint of its identity and characteristics. The emergence of MDR strains is invariably linked to alteration in the genomic configuration of an organism. This property is capitalized on to design genotypic biosensors that identify segments of DNA that impart resistance. Table 1 lists the differentially expressed or altered genes in MDR strains. 

Gene sequences used to identify MDR targets may be of two types: resistance imparting genes and mutated genes. The former is absent in drug-sensitive microbes and is present in resistant variants. For example, the mecA gene is present in MRSA but absent in MSSA (Methicillin Susceptible *S. aureus*). Watanabe et al. designed a biosensor capable of discriminating MRSA from normal *S. aureus* based on the presence of the mecA gene to a limit of 10 pM. This system was suited for on-site analysis because it did not require a thermocycler for the amplification of the target DNA [43]. Similarly, a capacitive label-free DNA sensor was devised by Liu et al. to detect the ampR gene in real-time from field-sourced samples. The device exhibited a limit of detection (LOD) in the picomolar range, exhibiting high sensitivity. Additionally, it was reusable, simple, and portable based on the capacitance drop across the sensor surface due to the hybridization reaction between the target DNA and oligonucleotide probe functionalized over the gold electrode [44]. An electrochemical sensor was designed to detect the NDM 1 gene in clinical samples with multifold sensitivity (LOD: 0.042 pg/L) by Zhang et al. The biosensor developed was capable of detecting single base pair mismatches in complex bacterial clinical samples without requiring any PCR amplification [45]. 

**Table 1:** MDR related genes in microorganisms.

Gene	Form Associated with MDR	Function	Resistance Against	Reference
mecA	Normal	Codes for alternative penicillin-binding protein PBP2a	Methicillin, nafcillin, oxacillin, and cephalosporins	[46]
rpoB	Mutated	Codes for the β-subunit of RNA polymerase	Rifampicin and isoniazid in multidrug resistant *Mycobacterium tuberculosis*	[47,48,49]
ampR	Normal	Involved in β-lactamase transcription, a transcriptional activator of the lysR family	3rd generation cephalosporinase	[50,51]
katG	Mutated	Codes for the catalase-peroxidase enzyme.	Isoniazid in *M. tuberculosis* when loss of function of the gene is seen	[52,53]
gyrA	Mutated	Codes for GyrA protein or DNA gyrase, a target of quinolones	Quinolones	[54,55]
inhA	Mutated	Codes for enoyl-ACP reductase of type II fatty acid synthase. which is crucial for the biosynthesis of mycolic acid (a component of the cell wall of *Mycobacterium*)	Isoniazid	[56,57]
hlyA	Normal	Codes for extracellular hyaluronate lyase Codes for α-hemolysin Shows enhanced virulence	--	[58,59]
YMDD motif in reverse transcriptase	Mutated YMDD motif	Locus/motif present in RNA-dependent DNA polymerase.	Lamivudine in *Hepatitis B virus*	[60,61]
K13 gene	C580Y mutation	Codes for Kelch protein	Artemisinin in *Plasmodium falciparum*	[62]
NDM1 gene(blaNDM-1)	Normal and variants	Codes for New Delhi metallo-β-lactamase-1 (NDM-1)	Resistance to carbapeneme and β lactam antibiotics (except for azetreonam)	[45,63]
gliT gene	Normal	Codes for gliotoxin A virulence factor associated with invasive aspergillosis	--	[64,65]

The second type of target genes for MDR identification fall into the mutated category. The mutations in the genes of these species result in the expression of targets (receptors, proteins, or other biomolecules) in a form that is not recognizable by AM drugs. For example, rifampicin and isoniazid fail to act on MDR *M. tuberculosis* due to the presence of the mutated rpoB gene [47,48]. The unmutated rpoB codes for the β-subunit of RNA polymerase targeted by these two drugs [49]. However, the mutation in the rpoB gene brings about structural/conformational changes in the enzyme, which significantly lowers the binding affinity towards these drugs. An electrochemical sensor with LOD in the femtomolar range was designed by Haddaoui et al. to detect mutated rpoB genes in MDR-*M. tuberculosis* (LOD: 4 fM). This DNA sensor was based on a nanocomposite of magnetic polypyrrole/Fe_3_O_4_ tagged with naphthoquinone on PAMAM which could discriminate the wild type rpoB gene, in drop-sized samples (50 µL sample or 3 × 10^4^ copies of DNA), from the mutated one [66]. Another gene mutation used as a target for biosensor development is the C580Y mutation in *Plasmodium falciparum*, which imparts resistance against choroquinone and artemisinin [62,67]. To this effect, Malpartida-Cardenas et al. developed a sensitive electrochemical sensor capable of detecting a single gene copy variation in less than 25 min. This was the first complementary metal oxide semiconductor-based lab-on-chip design biosensor for quantitative evaluation mutations in unknown *P. falciparum* samples [68].

Genes that impart enhanced virulence are also good targets for detecting MDR strains. For example, the hlyA gene, encoding α-hemolysin, has also been considered a target analyte, as its presence can be correlated to MDR in many pathogens [69]. An optical biosensor developed by Shi et al. provides visual detection and quantification for the hlyA gene in *Listeria monocytogenes* to a limit of 10 CFU/mL. The device combined loop-mediated isothermal amplification with propidium monoazide which enabled it to distinguish between viable and dead cells, and nanozyme which enabled visualization and imparted specificity and stability to the sensor [70]. Likewise, existing evidence suggests that the presence of the gliT gene correlates with invasive aspergillosis—a complication usually seen in immunocompromised patients [64,65]. A highly selective genotypic nano-biosensor was made using 1,6 hexane dithiol and chitosan stabilized AuNP for detecting the gliT gene by Bhatnagar et al., with a dynamic range of 1 × 10^−14^ − 1 × 10^−2^ M. This was the first biosensor ever developed for gliT detection sourced directly from fungal strains (LOD: 0.32 ± 0.01 × 10^−14^ M) [71].

There can be instances when a single analyte might not yield the required sensitivity or selectivity in detecting MDR strains. For such situations, biosensors based on multiplexing several analytes have the potential to enhance the sensitivity and selectivity limits. An example of such a biosensor developed by Dhar et. al. encompasses the simultaneous visual detection of point mutations in the rpoB, katG, and gyrA genes in MDR *M. tuberculosis* using split deoxyribozyme cascade probes [72]. Similarly, Bengtson et al. developed a multiplex biosensor to detect mutated rpoB, inhA, katG, and gyrA genes, and mutations in 23S rRNA, in MDR *M. tuberculosis*. This biosensor utilized a universal substrate significantly reducing the overall cost, had a high sensitivity compared to other devices utilizing molecular beacon probes, and could identify point mutations in both DNA and RNA [73].

Some biosensors analyze the whole DNA and/or RNA to determine the drug-resistant status of pathogens. An example of such a device is a duplex SPR-based biosensor designed to detect *M. tuberculosis* and pathogenic *E. coli*. (LOD: 5 nM). This sensor amalgamated the multiplexing ability and sensitivity of electrochemical and SPR-based sensing, with the robustness and selectivity of structure-switching nanomaterials [74]. Another electrochemical DNA-based biosensor capable of detecting point mutations to femtomolar limits in PCR samples of M. tuberculosis was developed by Bizid et al. This biosensor made use of a novel redox polymer, oligo-methoxy-phenyl-acetonitrile, deposited over a gold electrode, obtaining an LOD of 0.2 fM [75]. Tsao et al. used RNA instead of DNA to assess the MDR status of the influenza virus using peptide nucleic acid as both a sensor probe and a PCR clamp. This biosensor was sensitive to a concentration of 10 copies /mL of RNA from resistant viruses among 2 × 10^4^ copies of RNA from non-resistant influenza viruses [76].

#### 2.4.2. Phenotypic Biosensors for MDR Microbes 

The underlying basis of phenotypic biosensors is the detection of (a) MDR microbes, (b) MDR-associated analytes, and (c) biofilms. MDR microbes exhibit pronounced changes in cell phenotype compared to the normal strains, which can be capitalized on to determine the drug resistance status of the cell. For instance, Zheng et al. developed a multiplex fluorescence carbon dot array-based biosensor that could discriminate six different bacterial strains with a high accuracy and classify them according to their gram status. The dots were functionalized with polymyxin, vancomycin, and boronic acid, allowing bacterial discrimination with 91.6% accuracy [77]. A fluorescence-based duplex biosensor was used to detect MRSA and *Klebsiella pneumoniae carbapenemase 2-expressing Klebsiella pneumoniae* to a limit of approximately 20 CFU/mL in real-time, using a dual platform made of a broad-spectrum fluorescent opsonin probe and specific aptamer coated magnetic beads [78]. An electrochemical sensor system developed by Gill et al. for detecting MRSA employed porous copper nanocomposites modified with vancomycin, which could be used for both detection and as a theranostic tool. This was the first time a copper nanocomposite was investigated for its potential use in the treatment of MRSA infection and it yielded satisfactory outcomes (LOD: 5 CFU/mL and MIC: 1.93 μg/mL) [79].

Another class of sensors detects the presence of analytes linked to virulence and the MDR status of the cell. Detection of the presence or activity of antibiotic-inactivating enzymes like ß-lactamases, AmpC, extended-spectrum ß-lactamases (ESBLs), carbapenemases, and cephalosporinases can also be used to confirm the drug resistance status [80,81,82]. One such example is the fluorescence-based sensor with the ability to identify ceftazidime-resistant bacteria that was developed by Thai et al. to detect various ESBL producers to a limit of 10 CFU/mL in 90 min irrespective of their genotype [83]. 

A significant difference in cell wall composition was observed in the susceptible and MDR variants of *M. tuberculosis*. An ultrastructural analysis of the cell wall of the two strains revealed that the resistant strains had a sturdier cell wall, with a higher triglyceride concentration [84]. Mannose-capped lipoarabinomannan is an amphipathic lipopolysaccharide found in all *Mycobacterium* species, with a critical role in its survival and pathogenesis [84]. A plasmonic fiber optic absorbance biosensor was designed to detect this particular molecule in bacterial samples to a femtogram/mL limit [85]. Endotoxins are liposaccharides associated with MDR and are present on the outer leaflet of most Gram-negative bacteria [86]. For example, L-Ara4N modified Lipid A component of gram-negative endotoxins has been known to impart polymyxin resistance [87]. Yeo et al. developed a gold electrode-based electrochemical biosensor to detect endotoxins in *Escherichia coli* to a limit of 0.0002 EU/mL [88]. A decreased expression of pyocyanin, a secondary metabolite and virulence factor, is seen in MDR *P. aeruginosa* [89,90]. An electrochemical biosensor has been developed to detect pyocyanin by Rashid et al. in matrices of varying complexities like PBS, saliva, and urine samples to micromolar concentrations, using a combination of AuNP and reduced graphene oxide [91].

**Figure 4:**
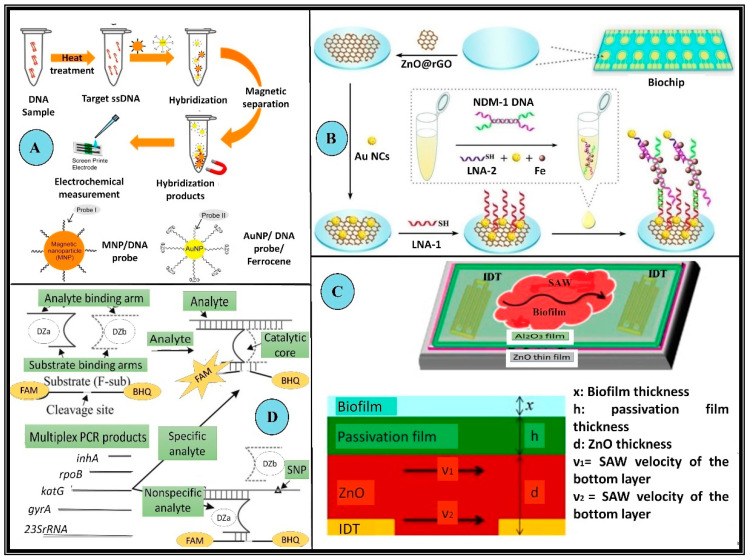
Examples of biosensors employed in the detection of MDR microorganisms. (**A**) Schematic illustration of target DNA detection using the DNA sensing system with modified Au nanoparticles and magnetic nanoparticles to detect MRSA. These microbes possess the mecA gene which makes these variants resistant to the actions of methicillin, nafcillin, oxacillin, and cephalosporins. In this setup first, the ssDNA sample is prepared using heat treatment, which is allowed to hybridize with modified nanoparticles (Au nanoparticles and magnetic). The hybridized products are then collected via magnetic separation followed by chronoamperometric detection (**B**) Schematic representation of a modified 2 × 8 array biochip detecting NDM 1 resistant bacteria. These bacteria produce a special class of β-lactamases which impart enhanced resistance to the microorganism. The biochip was made using Au NC@LNA-1/NDM-1DNA/LNA-2 Au NC in a “sandwich-like” model. (**C**) Schematic and a cross-sectional of the inverted passivated SAW sensor for the detection of biofilms. The piezoelectric surface is decorated with an interdigitated transducer. The relative thickness of all the layers and the velocity of waves along the piezoelectric surface is crucial to the design. (**D**) Schematic diagram of the binary deoxyribozyme (BiDz) sensors for multiplex AMR gene detection of XDR *M. tuberculosis.* DNA strands Dza and Dzb reform a deoxyribozyme catalytic core by hybridizing to the adjacent regions of the analyte F-sub, a fluorescent substrate containing a 3′ black hole quencher (BHQ) and 5′ fluorescein (FAM) label. When in proximity, these emit low fluorescence signals. When the substrate is cleaved, the fluorophore is separated from the quencher allowing for an increase in fluorescence. This BiDz sensor, when paired with the specific analyte, forms the catalytic core, which cleaves F-sub and produces a fluorescent signal. Alternatively, in the presence of a mismatched (nonspecific) analyte, the catalytic core of the sensor cannot be formed and the fluorescence of F-sub remains quenched *(All reproduced with permission)*.

The formation of biofilms is a prominent feature of various drug-resistant pathogens and chronic infections; however, their detection by conventional methods is non-trivial [92]. Specific biosensors have also been devised to detect biofilms. For instance, Kim et al. developed a surface acoustic wave-based sensor for detecting *E. coli* biofilms to picogram limits. The device demonstrated an amalgamation of bacterial biofilm sensing and treatment on a single chip [93]. Figure 4 gives a schematic overview of a variety of biosensors for MDR detection. The different features and specific design characteristics of many biosensors directed at detecting multidrug resistance in microbes have been extensively listed in Table 2. 

## 3. Multidrug Resistance in Malignant Systems and Biosensors

### 3.1. Foundation and Emergence of MDR in Cancer

Though MDR in cancers is a cause for concern, the underlying principles of its development are poorly understood. Compared with MDR in microbial cells, MDR in cancers is more enigmatic owing to its highly multifactorial nature [132]. Making things worse is the fact that not all facets contributing to MDR development in cancers are known completely [133]. The significant patient-to-patient heterogeneity in characteristics of MDR tumors makes its diagnosis a difficult task. 

Resistance may be innate (present before the subject is exposed to the drug) or acquired (during therapy) [133]. Innate chemoresistance results from genetic mutations, activation of pathways against xenobiotics, and tumor heterogeneity owing to pre-existing insensitive cell subpopulations [134]. Acquired chemoresistance, on the other hand, results from activating protooncogenes, altered expression of drug targets or mutations in them, or alterations in the tumor microenvironment (TME) [134]. While these factors invariably are linked with the emergence of MDR tumors, there are specific basic mechanisms to resist the actions of anticancer drugs. These include (a) preventing the drug from reaching its target, (b) effectively decreasing the drug concentration, (c) deploying compensatory mechanisms promoting survival, or (d) promoting dormancy in these cells [7,135]. 

### 3.2. Biochemical Basis of MDR in Cancer

**Figure 5:**
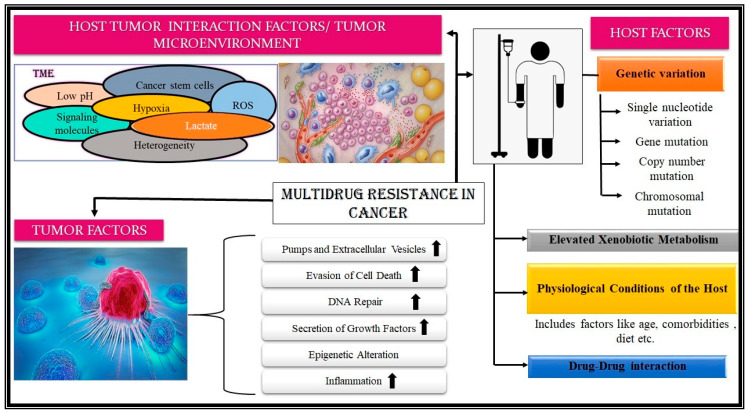
Factors contributing to the development of multidrug resistance. The factors leading to the development of MDR in cancers can be grouped into ‘Host associated’, ‘Tumor associated’, and ‘Host-Tumor interaction associated’ factors. Host-associated factors are factors associated specifically with a particular patient/subject. These include genetic variation, ranging from point to chromosomal mutation; enhanced xenobiotic metabolism; drug-drug interaction; and physiological conditions. Tumor factors are the features of the tumor that facilitate the development of multidrug resistance in cancers. These include enhanced cell growth, inflammation, DNA repair, evasion of cell death, epigenetic alterations, etc. The last contributing set of factors, i.e., Host-tumor interaction-associated factors, are where the confluence of various factors produces a niche to promote MDR development. Factors like perennial inflammation of the TME, surfeit of lactate, acidic pH, lack of oxygen, etc., in the TME are some of them. (The figure has been designed taking reference from the following resources: [2,10,29,30,33,76,78,80,81,82]).

While cancer is a widely studied disease, a complete understanding of its pathophysiology is still lacking. Cancer, when coupled with MDR, becomes even more challenging to understand. This complexity results from a cumulative effect of several mechanisms which are in constant crosstalk with one another [18]. The variability in tumor characteristics exhibiting MDR can be attributed to the differential interaction of these mechanisms across patient groups. A detailed account of all the contributing factors is beyond the scope of this review. However, the following references are excellent resources for gaining a comprehensive picture of the governing factors [2,10,30,31,36,132,134,136,137]. Figure 5 aims at summing up various factors responsible for MDR development in cancer cells.

The primary causative factor responsible for MDR in cancer cells is the differential functioning of the drug efflux pumps, belonging to the superfamily of ABC proteins. These ubiquitous transmembrane proteins catalyze the ATP-dependent transport of drugs [138]. The P-glycoprotein (Pgp) was the first efflux protein associated with MDR [10,85,139]. Later MRP1, BC RP, ABCG2/MXR, and others were recognized for their contribution to the development of MDR [84]. For a simplified overview, factors leading to MDR in neoplasm can be divided into host-associated, tumor-associated, and host tumor-interaction-associated factors [132]. 

#### 3.2.1. Host-Associated Factors

These factors include genetic variations seen in the host, the differential expression of MDR-associated proteins and pumps, variation in the metabolism of drugs, the physiological state of the patient, the different drugs prescribed in due course, its pharmacological profile, among others, which heavily contribute to intrinsic drug resistance among cancer patients [9,76,78,82,132,134,137]. Genetic variations, ranging from a single point or nucleotide variation to chromosomal alteration, accounts for 20–95% of the variability observed [132]. Aneuploidy is a common phenomenon observed in cancer cells. It is hypothesized to be a prominent reason for MDR as it may lead to the deletion of essential genes which had a role in drug response [137]. Gene mutation may lead to target modification or surpassing, enhanced expression of enzymes like cytochrome P450 and glutathione-S-transferase for exogenous drug/xenobiotic metabolism [140]. Polymorphism due to ethnicity has also been acknowledged in MDR-associated genes [141]. The physiological status of the host, i.e., age, nutrition, and the presence of comorbidities, also plays a critical role in the development of MDR [140]. The drug regimen a patient is prescribed also plays a decisive role in the development of MDR because of the possibility of drug-drug interactions, as patients are generally given several drugs and supplements concomitantly [140].

#### 3.2.2. Tumor-Associated Factors 

These alterations made by tumor cells are conducive to the development of MDR. These include enhanced expression of drug efflux pumps and extracellular vesicles (EV), evasion of cell death, dysregulation of DNA damage and repair machinery, epigenetic changes, secretion of growth factors, metabolic alterations, and promotion of heterogeneity in structure [134,139,140]. Drug efflux pumps effectively decrease the intracellular drug concentration, preventing its deleterious effects [132]. EVs are specialized bodies, which serve as notable vehicles of MDR dissemination as they help in extracellular drug trafficking and sequestration [142,143]. The evasion of apoptosis by manipulating pro and anti-apoptotic genes, and avoiding anoikis and autophagy are important strategies for MDR promotion [7,9,144]. An increased DNA repair rate and secretion of growth factors such as IL-6 (a potent promoter of chronic inflammation) also accelerate MDR development [137]. 

#### 3.2.3. Host-Tumor-Interaction Associated Factor

A tumor cell usually promotes the maintenance of the malignant phenotype due to intricate interactions between the host and the tumor. These interactions between the host and tumor form an ecological niche called the TME. The TME is formed by physiological and cellular (malignant and non-malignant type) constituents enabling the neoplasm to thrive and progress [145,146]. The appropriate intra-tumoral heterogeneity, and ample genetic and epigenetic diversity, allow the therapy-induced expansion of pre-existing drug-resistant cancer cell clones in the space, promoting chemoresistance [9,132,140,145]. Cancer stem cells play a crucial role in MDR development, efficient seeding of cancer upon metastasis, and enhancing the plasticity of the neoplasm [136,147,148]. This environment is notably acidic (pH 6.3–6.9); hypoxic; perennially inflamed; and filled with poorly formed vasculature, reactive oxygen species, growth factors, and signaling molecules (which promote inflammation, immunosuppression, and tumorigenesis); and has a surfeit of lactate [149,150]. These conditions produce a selective pressure which leads to the propagation of resistant cells as well as providing a haven from antineoplastic agents.

### 3.3. Use of Biosensors in Detecting MDR in Neoplasms 

For this review, the MDR-detecting biosensors for cancerous cells have been divided into *genotypic, phenotypic,* and *drug pharmacokinetics-based* biosensors.

#### 3.3.1. Genotypic Biosensor for MDR Cancer 

Biosensors targeting genotypic traits detect any discrepancy in the cell’s genetic makeup that may result in the development of resistance. Peng et al. designed an electrochemical biosensor for detecting MDR1 using an AuNP/toluidine blue-graphene oxide-modified electrode (LOD: 3.12 fM) [151]. Further, Chen et al. made a label-free and enzyme-free biosensor with improved sensitivity to detect the same gene in clinical leukemia samples using nitrogen-doped graphene nanosheets functionalized over AuNP (LOD: 2.95 pM) [152] Graphene and related materials exhibit suitable optical, electronic, and electrochemical characteristics, making them an excellent choice for versatile fabrication [153]. Xiang et.al. developed a multiplexed fluorescence-based biosensor that could simultaneously detect seventeen different drug-sensitive and drug-resistant mutations in cell-free circulating DNA in real-time to a detection limit of 1–4 copies [154]. This biosensor identified the T790M mutation in the epidermal growth factor receptor (EGFR) gene, which makes it resistant to drugs like gefitinib and erlotinib [154,155].

Another highly sought-after method of MDR analysis is detecting and monitoring micro-RNA (miR) levels. miRs are short, non-coding, and highly conserved RNA moieties that function in post-transcriptional gene regulation via translational repression and mRNA degradation [156]. miRs may be expressed constitutively or produced only under specific circumstances differentially [157]. Fluctuations in miR levels in the case of MDR variants are described in Table 3. Hence these molecules are ideally suited to detect MDR in malignant cells. Cai et al. developed a microfluidic-based laser-induced fluorescence sensor to determine miR-21 and miR-31 levels in HeLa and A549 cell lysates in real-time, with an LOD of 0.20 fM and 0.50 fM for miR-21 and miR-31, respectively [158]. Yaman et al. developed disposable AuNP peptide nanotubes-based biosensors for detecting miR 410 in serum and prostate cancer cell lines. In minutes, these impedimetric biosensors could detect the miR to the femtomolar limit [159]. It was observed that major feats have been achieved in the development of fluorescence-based biosensors in the detection of miRs This can be attributed to such biosensors exhibiting fast response, multiplexed ability, having simple instrumentation and operation, high selectivities and specificities, a non-destructive nature, and they can be easily integrated with microfluidic platforms [21,160].

#### 3.3.2. Phenotypic Biosensor for MDR Cancer

Perceptible changes in the cells accompany the MDR of the neoplasm. These changes may include some molecules being over or under-produced compared to usual, some significant changes in the TME that may be aiding in the development of resistance, an overall change in the cellular profile, and the production of anomalous molecules. Sometimes even the behavior or the overall shape becomes strikingly distinguishable, which aids in the detection of MDR cell lines. Specific biosensors are designed to detect and monitor these phenotypic changes to know about the status of the cell concerning drug resistance. 

A biosensor was developed by Hu et al., which was based on the difference in electrochemical behavior as exhibited by drug-sensitive versus drug-resistant leukemia K562 cell lines to a limit of 500 cells [190]. Since the resistant and non-resistant cells have significant differences in their PM profiles, it serves as an avenue for discernment. Tao et al. developed sensing systems on Au-nanoclusters that could efficiently discern drug-resistant, cancerous, metastatic, and healthy human breast cancer cells to a limit of 200 cells, based on the unique chemistry and surface charge densities of each type of cell [191]. 

One of the most popular candidates for the detection of MDR is the Pgp. For instance, Chandra et al. designed an amperometric biosensor using monoclonal antibodies for Pgp as a target analyte. This sensor could detect MDR lines to an LOD of 2372 cells/mL in MDR_CC_ cell lines [192]. Gulati et al. developed an optical biosensor with higher sensitivity, capable of detecting Pgp at concentrations as low as 27 cells/mL [193]. Another drug efflux pump that can be used to assess the MDR status is the MRP2/ABCC2 protein. Li et al. developed a microfluidics-based biosensor that was sensitive enough to detect this protein in a single cell based on hydrodynamic theory [194]. 

Specific proteins are overexpressed in the case of MDR cancer cell lines, to promote tumor survival (Figure 5). For example, the enhanced expression of β1 integrin, a membrane receptor, helps anchorage to the extracellular matrix and plays a role in anoikis correlated to increased resistance [195,196,197]. Jiang et al. developed an electrochemical immunosensor using a mouse anti-human monoclonal antibody functionalized over a glassy carbon electrode to detect β1 integrin to a limit of 3.5 × 10^3^ cells/ mL [198]. Drug-resistant neoplastic cells are also known to exhibit upregulation of complement regulatory proteins. These proteins are present on the membranes of normal cells, serving as a survival strategy to prevent complement system activation. However, when expressed at abnormally high amounts in tumor cells, they lead to its evasion of attack by the complement system [199]. Choudhary et al. developed an immunosensor for early non-invasive detection of oral cancer from clinical saliva samples by detecting CD59, a complement restriction factor [200]. Brain-derived neurotrophic factor (BDNF) is a class of neurotrophins associated with decreased sensitivity to cisplatin, etoposide, and vinblastine in numerous cancers [201]. Akhtar et al. developed an ultrasensitive biosensor to detect the levels of BDNF in serum samples. This sensor was further used to study and monitor the effects of various activators of BDNF producers, like nicotine and alcohol, in the model cell lines SH-SY5Y and PC-12 [202].

Enzymes are common biomarkers that are used for the detection of MDR in cancer cells. For instance, a fluorescence-based biosensor was developed by Wang et al. to measure the real-time intracellular telomerase activity in A549, HepG2, and MCF7 cell lines [203]. Increased telomerase activity, or its upregulated expression, is a feature of cancer cells resistant to drugs like cisplatin and 5-fluorouracil, as it regulates the reactive oxygen species and imparts resistance against drugs targeting ABC transporters, DNA damage, and apoptosis [204]. Another repertoire closely related to the MDR profile is glutathione and associated enzymes. Overexpression of glutathione S-transferase has been noted in MDR cell lines. Glutathione is required as a co-factor for efflux protein ABCC1 activity [137,205,206,207,208]. A fluorescence-based biosensor was developed by Yang et al. for glutathione monitoring in chemo-resistant cell lines such as HeLa and HepG2 to a limit of 87 nM. This was the first biosensor developed for imaging glutathione levels using a two-photon nanoscale metal-organic framework [209]. DNA methyltransferases cause methylation of regions of DNA. Hypermethylation of gene segments, such as the promoter of glutathione S-transferase, is linked to higher levels of poor prognosis and MDR development [205,210]. A fluorescence-based sensor, employing a novel dumbbell-shaped DNA template for copper nanoparticles, was developed by Yin et al. to detect this enzyme to a limit of 0.16968 mU/μL [211].

The conditions in the TME promote MDR in tumors. Hypoxia and a highly acidic pH are two significant conditions prevalent in the TME. Nitro reductase is an endogenous enzyme expressed in highly hypoxic conditions [212]. Wang et al. developed a fluorescent probe for in vivo detection of the enzyme in the TME, with a dynamic range of 15–300 ng/mL and an LOD of 0.27 ng/mL [213]. To monitor the pH of the TME, a fluorescence-based biosensor was devised by Ge et al., responsive in the range of 4.2–6.4 (pKa = 5.18), capable of giving a readout in approximately 1 min [214]. 

#### 3.3.3. Drug Pharmacokinetics-Based Biosensors for MDR Cancer 

An indirect approach to detect MDR is by monitoring the pharmacokinetic parameters of the administered drug. The core principle of this method is based on the most common mechanisms a tumor resorts to for avoiding the adverse effects of a chemotherapeutic agent, which is the extrusion of drugs out of the cell. Monitoring the levels of the drugs in clinical samples may, therefore, serve as an indirect indicator of the MDR status of the cell. One such device was designed by Zhao et al. to detect methotrexate in serum and clinical samples. This SPR-based biosensor used folic acid functionalized AuNP and was sensitive to methotrexate to a level of 155 nM, yielding results in under 60 s [215]. A novel SER sensor design using cysteine-AuNP conjugate was developed to assess the levels of exosomes by Hunter et al. [216]. Exosomes are a subset of EVs used for transferring bioactive molecules between cancers and various destinations within and beyond the TME, helping in the development of increased chemoresistance by methods like mediating drug efflux, neutralization of antitumor antibodies, and carrying resistance imparting miRs to drug-sensitive cells [143,217,218]. This biosensor was able to detect exosomal cis-platin and exosomes to a limit of 0.17 µg/mL and 65 nM, respectively, with an accuracy of >90% in diagnosis [143,216,217,218]. 

**Figure 6:**
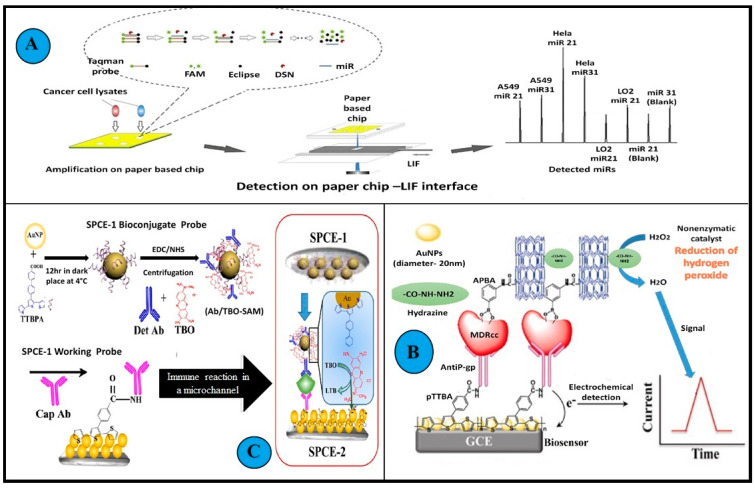
Examples of biosensors used in the detection of MDR cancer cell lines. (**A**): A graphical abstract of amplification and subsequent detection of miRNA in cancer cells using a microfluidics paper-based fluorescent sensor. A confocal LIF detector was used for the determination of miRNAs on microfluidics paper. An interface was designed and applied to obtain a stable fluorescence signal. DSN amplification on a double-layer microfluidics paper was performed to improve the sensitivity of the system min. (**B**): Illustration depicts the working of immunosensors detecting BDNF. Two screen-printed carbon electrodes (SPEC) with long microfluidics channels were assembled. This sandwich immunosensing approach helped in detection by generating signals using redox indicator TBO. First an Ab-Ag complex was formed on the working probe, succeeded by the Ag and Lyophilized BDNF antibody Det Ab reacting with the biconjugate probe. (**C**): Schematic representation of the amperometric immunosensor fabrication and detection principle of drug-resistant cancer cells in biological matrices. The unit composition included a modified glassy carbon electrode, gold nanoparticles, polymeric TTBA (2,2′:5′, 2″-terthiophene-3′(*p*-benzoic acid) (TTBA)), and anti Pgp monoclonal antibodies. The MDR cancer cells are captured in between this engineered layer and APBA (amino phenyl boronic acid), multi-walled carbon nanotubes, and hydrazine conjugate. (All reproduced with permission).

Some of the examples above are illustrated in Figure 6, while Table 4 comprehensively discusses multiple biosensors which aim at detecting MDR in malignant systems. 

## 4. Discussion and Future Perspectives

In this review, we have discussed the details of the foundation and emergence of MDR and the mechanisms by which malignant and microbial systems achieve MDR. Further, various aspects of the pathophysiology that serve as unique signatures of MDR are suitably utilized in designing different biosensors. In this section we shall discuss some pressing questions:

### 4.1. What Kind of Threat Does MDR Pose?

With the ever-increasing use of AM antibiotics in farming, healthcare, excessive prescription of antibiotics by clinicians, availability of antibiotics over the counter in many parts of the world, and negligence on the part of the patients coupled with globalization and population explosion; drug resistance can be perceived as the next emerging global health concern [4,233,234]. According to the latest projections, drug resistance (microbial) related deaths are projected to reach 10 million by the year 2050, amounting to a global economic burden of a staggering 100 billion USD. In 2021, the bacterial multidrug resistance market is valued at 10.359 billion USD, which is expected to reach 16.02 billion USD by 2029 [235]. For instance, the statistics of MDR TB may be considered as an example. Presently, 41% of newly reported cases of the disease are of the multidrug-resistant type [236]. In 2020, cancer accounted for nearly 10 million deaths [237]. Nevertheless, the WHO states that 30–50% of cancer cases can be managed by risk factor avoidance, early detection, and treatment strategies. Selective large-scale screening and early diagnosis are two pillars of detection [238]. Hence it becomes crucial to search for methods to tackle MDR, as cases associated with drug resistance increase exponentially with each passing year.

### 4.2. What Is the Current Scenario of Diagnosis?

As discussed previously, current approaches to MDR detection are primarily achieved using conventional techniques. Though these techniques offer credible results, they suffer from long turnaround times, rendering mass screenings a tedious task. Furthermore, it is difficult to scale conventional methods of MDR detection owing to the requirement of a skilled workforce for sample preparation, processing, and the interpretation of results. In addition to this, since the clinical manifestation of cancers often occur at later stages of progression, the conventional approaches fail to provide an early diagnosis of the disease as they rely on the phenotypic assessment. 

### 4.3. How can Biosensors Be Used to Address These Problems?

Biosensors can potentially serve as a sturdy ancillary panacea to this problem. Quick turnaround times with high sensitivities, specificities, linearities, and robustness, along with ease of operation make them suitable to be used as POC devices for MDR detection [33,239,240]. These can be used in biomarker detection in a variety of sample matrices, tumor diagnosis and characterization, and medical imaging. An ideal biosensor for MDR detection is expected to differentiate resistant cell types vs. normal cells, and to give insights into the susceptibility of the cells and host immune response [33,160,240]. As is evident, most of the biosensors discussed herein are based on electrochemical or optical detection. Such biosensors offer excellent stability, reliability, robustness, compactness, and could be used for developing multiplex systems by exploiting surface chemistry, nanomaterials, and different transducing mechanisms (voltammetric, amperometric, capacitance-based, SPR, SER, fluorescence, etc.) [23]. Further, microfluidics-based sensors render the analysis of DNA damage, cytotoxicity, and detection of pathogens easier, as they can easily assimilate and define the biochemical microenvironment seamlessly both temporally and spatially [160]. These are major technological leaps that have made the development of lab-on-chip models possible [160,235,241]

The ease of reliable data generation using such sensors can add a new dimension to integrating data science into the study of MDR. Recent studies have demonstrated the use of machine learning models and algorithms to predict MDR trends in *M. tuberculosis* and urinary tract infections [242,243]. Some of the sensors discussed in this review have also been tested for their theragnostic abilities, hence the scope of biosensors is not limited to only diagnosis. It has been well established that a significant cornerstone for MDR prevalence is linked to the indiscriminate use of AM and its accumulation in the environment. To curtail the dissemination of MDR, many sensor systems have been designed to carefully monitor the levels of drugs and antibiotics in clinical and environmental samples. The urgency to resolve this problem has led to the development of some innovative systems that not only detect the cells but also monitor their growth. 

### 4.4. Possible Roadblocks and Shortcomings

However, for the complete realization of biosensors’ potential, or establishing them as a valid alternative to conventional testing, several bottlenecks need to be overcome. On the designing front, work needs to be done concerning minimizing the matrix effect and system integration [239]. A disadvantage of many biosensors is the requirement of processing samples, which makes them difficult to operate [239]. A possible solution to overcome this issue would be to develop biosensors that could yield data non-invasively using saliva, sweat, and urine samples. Another focus area that needs work is the miniaturization and portability of devices and making them more user-friendly. For successful translation from a laboratory prototype to an end-user commercial product, a high capital investment is required in the research and development of such devices and for the subsequent rounds of evaluation and approval by regulators. This leads to an extremely slow rate of commercialization of biosensors [239].

## 5. Conclusions

MDR is a pressing scourge on the healthcare system, and the need to develop efficient solutions is imminent. While significant technological advancements have taken place in terms of the development of biosensors that can help MDR detection in complex matrices and a plethora of clinical indications, we are still far away from replacing conventional detection strategies with a biosensors-based approach. Nevertheless, with the rapid advancements in our understanding of the pathobiology of various diseases, many new biomarkers have been discovered that have huge potentials for clinical translations. This, coupled with novel biosensor systems, significantly enhances the prospect of the early diagnosis and management of MDR cancers. 

## Figures and Tables

**Table 2:** Biosensors used to detect MDR in microbial systems.

S.No.	Microorganism(s) (M/O) Detected	Target Analyte of the Biosensor	Method of Detection	Linearity	Limit of Detection	Response Time	Salient Features of the Biosensor Design or Principle	Reference
**I. Genotypic Biosensors for MDR Microbe Detection**								
1	Multiple M/O	ampR gene	Electrochemical	1 pM to 1 nM	<1 pM (ss ampR) 4 pM (ds ampR)	20 min	Capacitive DNA biosensor Label free Probe functionalized electrodes reusable for at least 6 cycles.	[44]
2	*Staphylococcus aureus*	mecA gene	Electrochemical	10 to 166 pM	10 pM	--	Selective for MRSA and *S. aureus* 2 types of nanoparticle-modified probes were used. Linearity observed from 10–166 pM	[43]
3	*S. aureus*	mecA gene	Electrochemical	0.075 to 200 pM	63 fM	2 h	Isothermal strand-displacement polymerization reaction based Methylene blue hairpin probes used on gold electrodes	[94]
4	*M. tuberculosis*	rpoB gene katG gene gyrA gene	Optical	--	1.5–13 nM	1.5–2 h	Colorimetric detection system Deoxy ribozyme sensors used Point mutations in mentioned genes identified	[72]
5	*Listeria monocytogenes*	hlyA gene	Optical	--	10 CFU/mL	--	• Can distinguish between dead and viable cells. • Visual detection and quantification. • Also, able to detect *L. monocytogenes* biofilm (mentioned further)	[70]
6	*Aspergillus fumigatus*	gliT gene	Electrochemical	1 × 10^−14^ to 1 × 10^−2^ M	0.32 ± 0.01 × 10^−14^ M	≤20 min	• The self-assembled probes (gliP) were immobilized over Au electrodes. • Au nanoparticles were stabilized by 1,6-Hexanedithiol and chitosan.	[71]
7	*M. tuberculosis*	rpoB gene	Electrochemical	1 fM to 0.1 pM	4 fM or 3 × 10^4^ copies of DNA	--	• Polypyrrole-coated Fe_3_O_4_NPs functionalized with PAMAM dendrimers were used as scaffolds • The Naphthoquinone redox group and DNA probes were bound to the scaffolds	[66]
8	*Acinetobacter baumannii*	Carbapeneme resistance genes	Optical	--	--	<2 h	• Multiplex detection system • DNA extraction, PCR amplification, and silver nitrate-based colorimetric are essential components of the process.	[95]
9	*M. tuberculosis*	rpoB inhA	Optical	20 µg/mL to 50 µg/mL	30 µg/mL	30 min	• A duplex colorimetric detection system • Au nanoprobes used	[96]
10	*M. tuberculosis*	rpoB gene	Electrochemical	--	1 nM	--	• Thiolated DNA probes used • Impedimetric biosensor	[97]
11	*M. tuberculosis*	rpoB gene inhA gene gyrA gene katG gene 23S r RNA	Optical	--	--	--	• Binary deoxy ribozyme sensors used • Fluorescence-based multiplex detection system • Can also detect other mutations	[73]
12	*A. baumannii*	β-lactamase gene	Optical	10^2^ to 10^5^ CFU/mL	50 CFU/mL	<= 2hr	• PCR and CRISPR-CAS-based multiplex fluorescence-based detection system fabricated in array format	[98]
13	--	NDM1 gene	Electrochemical	1 pg/L to 100 μg/L	0.042 pg /L	1 min	• No PCR amplification is required • Sandwich-type LNA electrochemical biochips used • Detection carried out in clinical samples	[45]
14	*Escherichia coli* *Klebsiella pneumoniae*	NDM1 gene	Optoelectrical		100 copies	<3 min	• Thin Film Transistor sensors • Isothermal DNA amplification	[99]
15	*E. coli*	blaNDM-5 gene blaCTX-M15 gene	Optical	20 to 30 aM	25 aM	<30 min	• Microfluidic system • Bimodal waveguide interferometric biosensor detecting carbapenemase and ESBL encoding genes	[100]
16	*M. tuberculosis*	DNA	Electrochemical	1 fM to 100 pM	0.2 fM	--	• Can detect single point/nucleotide mutation	[75]
17	*M. tuberculosis* *E. coli*	DNA	Electrochemical	--	5 nM	Few minutes	• Duplex Surface Plasmon Resonance (SPR) sensing system • Can be extended to the detection of other biomolecules too	[74]
18	*E. coli* *K. pneumoniae* *P. aeruginosa* *S. aureus* *Enterobacter faecalis*	DNA	Optical	10^2^ to 10^3^ CFU/mL	4.5 CFU/mL	--	• Surface-enhanced Raman spectroscopy (SERS) based detection • Au-Ag core-shell nano dumbbells used	[101]
19	*S.aureus*	DNA	Electrochemical	--	100 fM	--	• An electrode made of reduced graphene oxide was used.	[102]
20	*M. tuberculosis*	DNA	Optical	10^−12^ M to 10^−8^ M	5 pM	--	• SER-based detection • Au nanoparticles modified probes • Enhanced surface-anchored rolling circle amplification employed • The positive mutation detection is achieved with a wild-type to the mutant ratio of 5000:1	[103]
21	*M. tuberculosis*	DNA	Electrochemical	--	~nM	--	• Able to detect single nucleotide substitution in folded NA structure	[104]
22	*Enterococcus*	DNA	Optical	--	10^2^ CFU/mL	45 min	• Colorimetric detection clubbed with loop-mediated isothermal amplification. • Vancomycin-resistant *Enterococcus* was detected.	[105]
23	*S. aureus*	DNA	Optical	--	10 CFU/mL	<20 min	• SPR-based detection • Distinguishes between MRSA MSSA and borderline oxacillin-resistant Staphylococcus aureus	[106]
24	*L. monocytogenes*	DNA	Electrochemical	--	--	--	• Single-stranded DNA probe immobilized over Au surface. • Changes in cyclic voltammetry peak current were recorded	[107]
25	*M. tuberculosis*	DNA	Optical	--	1µM	<20 min	• Label-free DNA detection and amplification	[108]
26	*M. tuberculosis*	DNA	Electrochemical	10^−18^ moL/L to 10^−14^ moL/L	0.330 aM	--	• G4-hemin used as an enzyme	[109]
27	*E. coli*	DNA	Electrochemical	10^−6^ to 10^−16^ M	0.1 fM	--	• Graphene oxide-nickel ferrite-chitosan nanocomposite film-based sensing platform	[110]
28	*S. aureus*	DNA	Diffusion based	10 to 60 pM	10 pM	10 sec	• Based on nanobead diffusometry and non-PCR-based DNA monitoring	[111]
29	*Salmonella* spp. *S. aureus* *E. coli*	DNA	Optical	--	3.0 × 10^2^ CFU/sample (Gram-negative) 3.0 × 10^3^ CFU/sample (Gram Positive)	<2 h	• Nucleic acid testing • Performs DNA extraction, polymerase chain reaction, and on-site colorimetric detection for point-of-care diagnosis • Multiplex detection system • DNA extraction and PCR amplification can be performed. • Colorimetric biosensor	[95]
30	*Plasmodium falciparum*	C580Y mutation	Electrochemical	--	1 copy/reaction volume	<25 min	• Potentiometric biosensor • Ion-Sensitive Field-Effect Transistors based lab-on-chip model.	[68]
31	*Influenza virus*	RNA	Optical	--	10 copies/mL of RNA	--	• Fluorescence-based detection • 10 copies/mL of RNA from the resistant strain among 2 × 10^4^ copies/mL of RNA from the sensitive strain	[76]
32	*Hepatitis B Virus*	DNA	Electrochemical	4 × 10^−10^ to 1 × 10^−8^ mol	1 × 10^−11^ mol	--	• Nonporous gold platform • Low cycles of PCR for amplification are required by the use of the Au platform	[112]
II. Phenotypic Biosensors for MDR Microbe Detection								
Phenotypic Biosensors Detecting the MDR Microbes Themselves								
33	*E. coli*	M/O	Optical	3.81 × 10^2^ to 2.44 × 10^4^ CFU/mL	460 CFU/mL	--	• Real-time detection in human urine, tap water, and apple juice • Colistin-modified carbon dots used • Fluorescence-based detection.	[113]
34	*S. aureus* *E. coli*	M/O	Optical	9 × 10^7^ CFU/mL	--	2 h	• Photoluminescence-based biosensor. • Graphene quantum dots based.	[114]
35	*E. coli*	M/O	Optical	10^5^–10^8^ CFU/mL	9.5 × 10^4^ CFU/mL	--	• Fluorescence-based detection • Water-soluble carbon dots used • Efficient in HeLa cell imaging	[115]
36	*E. coli* *Desulfovibrio desulfuricans* *S. sciuri* *L. monocytogenes* *S. aureus* *Pseudomonas aeruginosa*	M/O	Optical	--	--	--	• Multiplex detection and differential analysis of microbes • Carbon dots functionalized with 3 different receptors, boronic acid, polymyxin, and vancomycin, present on the fluorescence-based array • Discrimination of the six kinds of bacteria with 91.6% accuracy	[77]
37	*S. aureus* *E. coli*	M/O	Optical	10^1^ to 10^7^ CFU/mL	3 CFU/mL 3.5 CFU/mL, respectively	~2 h	• A multifunctional alternative current electro-kinetic SERS-based microfluidic system. • Can concentrate bacteria from whole blood, identify bacterial species, and determine antibiotic susceptibilities of the bacteria rapidly. • Label-free antibiotic susceptibility testing is possible with the device.	[116]
38	*S. aureus*	M/O	Optical	10 to 10^6^ CFU/mL	6.9 CFU/mL	--	• Bacteria imprinted film with N-Succinyl-Chitosan doping. • Fluorescence-based sensor • Au disulfide NP used.	[117]
39	*S. aureus*	M/O	Electrochemical	10 to 10^7^ CFU/mL	5 CFU/mL	30 min	• 3D porous copper nanocomposite modified with vancomycin was used. Also designed for the treatment of MRSA. • MIC:1.93 μg/mL	[79]
40	*E. coli*	M/O	Optical	5.0 × 10^1^ to 1.0 × 10^9^ CFU/mL	50 CFU/mL	--	• Enzymatic redox reaction employed • CD-MnO2 nanosheets are used as a platform • Label-free fluorescent biosensor • Considerable selectivity for *E. coli*	[118]
41	*S. aureus* *K. pneumoniae*	M/O	Optical	20 to 10^8^ CFU/mL	~20 CFU/mL	15 min	• Aptamer-coated magnetic beads used • A broad-spectrum fluorescent probe was used. • MRSA and *Klebsiella pneumoniae carbapenemase 2-expressing Klebsiella pneumoniae* (KPC-2 KP) can be detected • Crystallizable mannose-binding lectin-coated Au nanoclusters-based duplex detection system	[78]
42	*E. coli*	M/O	Optical	--	1.6 × 10^3^ CFU/min	10 min	• Optically induced electrophoresis phenomena are used to segregate resistant and non-resistant bacteria in a heterogeneous sample.	[119]
43	*S. aureus* *K. pneumoniae* *E. coli*	M/O	Optical	1 × 10^2^ to 1 × 10^6^ CFU/mL	67CFU/mL 57CFU/mL 61CFU/mL	4 h	• DNAzyme integrated with SPR system in the biosensor	[120]
44	*P. aeruginosa*	M/O	Optical	10^1^ CFU/mL to 10^7^ CFU/mL	9 CFU/mL	--	• Aptamers conjugated with photoluminescent carbon dots as probes • Graphene oxide is used as a quencher	[121]
45	*Salmonella infantis*	M/O	Optical	--	100 CFU/mL	1 h	• Anti-salmonella antibodies were adsorbed on single-walled carbon nanotubes • Field Effect Transistor (FET) based biosensor	[122]
46	*A. baumannii*	M/O	Optical	1 × 10^4^ to 5 × 10^7^ CFU/mL	2.3 × 10^3^ CFU/mL	--	• Diagnosis in sputum • Photoluminescent Au-Ag nanoclusters used	[123]
47	*S. aureus* *A. baumannii*	M/O	Electrochemical	--	10^4^ cells/mL	5 min	• Single-cell detection of antibiotic-resistant bacteria. • Voltametric biosensor	[124]
48	*S. aureus*	M/O	Optical	10^2^ to 10^7^ CFU/mL	33 CFU/mL	20 min	• IgY-modified immunosensor used. • Based on long-period fiber grating	[125]
49	*Candida albicans* *Cryptococcus neoformans*	M/O	Optical	0 to 2 µM	--	-	• Can also be used for Fe detection • Fluorescence-based biosensor • Uses N-doped carbon dots obtained from *Chionanthus retusus*	[126]
Phenotypic Biosensors Detecting MDR Associated Analytes								
50	20 different strains with extended-spectrum ß-lactamase (ESBL) activity	β-lactamase activity	Optical	--	10 CFU/mL	90 min	• BODIPY fluorescence-based probe was used • Can identify ceftazidime-resistant bacteria	[83]
51	*P. aeruginosa*	Pyocyanin	Electrochemical	1–100 μM	0.27 μM(PBS) 1.34 μM(Saliva) 2.3 μM(Urine)	--	• Reduced graphene oxide with Au nanoparticles used	[91]
52	*M. tuberculosis*	Mannose-capped lipoarabinomannan	Optical	5 fg/mL to 10 pg/ mL (PBS) 10 fg/mL to 10 pg/ mL (synthetic urine)	1–10 fg/mL	--	• A plasmonic fiber optic biosensor (P-FAB) strategy used	[85]
53	*E. coli*	Endotoxin	Electrochemical	0.0005 to 5 EU/mL	0.0002 EU/mL	--	• rhTLR4/MD-2 complex is the Bio-recognition Element (BRE) • Au electrodes used • High specificity	[88]
54	*M. tuberculosis*	MPT64 protein	Electrochemical	1 to 50 nM	81 pM	30 min	• Aptamers used as BRE • Gold electrode used	[127]
55	*K. pneumoniae*	Carbapenemase	Electrochemical	1 × 10^−12^ to 1 × 10^−7^ mol/L	0.2 pM	--	• Glassy carbon electrode modified with Au nanoparticles and graphene nanocomposite used	[128]
56	*Escherichia coli*	ESBL production	Optical	--	10^5^ CFU/mL	20 min	• β-lactamase activity monitored • CENTA used as β-lactamase reporter • SER-based paper biosensor	[129]
57	*S. aureus*	α-haemolysin	Optical	0.012 to 0.76 µM	0.002 µM	<30 min	• SPR-based system of detection using a cantilever system in combination with molecular imprinted gold chips. • Detection from septic blood samples	[130]
Phenotypic Biosensors Detecting Biofilms								
58	*S. aureus* *E. coli* *P. aeruginosa*	Biofilm	Electrochemical	--	10^4^ − 10^5^ CFU/cm^3^	--	• Monitored biofilm growth • Graphene oxide-based potentiometric biosensors	[131]
59	*E. coli*	Biofilm	Mechanical	--	5.3 pg	--	• An atomic layer deposition aluminum oxide sensor was used protected by ZnO • Surface Acoustic Wave (SAW) based detection	[93]
60	*L. monocytogenes*	Biofilm	Optical	--	1.164 × 10^1^ CFU/mL (stainless steel) 1.021 × 10^1^ CFU/mL (lettuce)	--	• Can distinguish between dead and viable cells. • Visual detection and quantification of the hlyA gene	[70]

**Table 3:** miRNA And Their Expression in the Case of MDR.

miRNA	Function	Expression Levels in the Case of MDR Phenotype	References
miR-21	Regulatory role in apoptosis, development, and differentiation of normal cells. Role in metastasis and carcinogenesis.	Upregulated	[161,162]
miR-155	Role in immune response, inflammation, and differentiation of hemopoietic lineages and tumorigenesis	Upregulated	[163,164]
miR-205	Regulates cell survival, proliferation, and susceptibility to chemotherapy	Downregulated	[165]
miR-122	Liver-specific miRNA. (70% of the liver’s miRNA pool)	Downregulated	[166,167]
miR-223	Haematopoetic cell-specific miRNA. Important for the development of cells in myeloid lineage	Downregulated	[168,169,170]
miR-31	Embryonic implantation and development, Muscle and bone homeostasis; Regulation of immune system function, and autoimmunity	Downregulated	[171]
miR-200a-3p	Inhibits malignant transformation and all stages of carcinogenesis	Downregulated	[172,173]
miR-34a	Tumor suppressor gene. Involved in the regulation of cell survival, migration, and remodeling properties	Downregulated	[143,174,175]
miR–k12-5-5p	Coded by Kaposi’s sarcoma (KS) associated with the herpes virus. Works in inhibiting replication and as a transcription activator	Upregulated (in metastasis and cell growth) and KS	[176,177,178]
miR-410	May promote or suppress tumor formation.	Downregulated	[179,180]
miR-196a-5p	Involved in metastasis	Upregulated	[181,182]
miR-141	Tumor suppressor gene	Upregulated	[183,184]
Let7 miR family	Roles in embryogenesis, tumorigenesis, development, and metabolism	Downregulated	[185,186,187]
Survivin	Inhibitor of apoptosis. Regulates cellular proliferation and death	Upregulated	[188,189]

**Table 4:** Biosensors used in the detection of multidrug-resistant tumors.

S.No	Target Analyte Detected by the Biosensor	Method of Detection	Linearity	Limit of Detection	Response Time	Cell Lines/Samples Used	Salient Features of the Biosensors’ Principle/Design	Reference
I. GENOTYPIC BIOSENSORS								
I.i Genotypic Biosensors Detecting Mutations or Gene Segments								
**1**	MDR1 gene	Electrochemical	1.0 × 10^−14^ to 1.0 × 10^−7^ M	3.12 fM	3.4 h	Clinical leukemic samples	Label-free biosensor N-doped graphene nanosheets functionalized over Au nanoparticle	[152]
**2**	MDR1 gene	Electrochemical	1.0 × 10^−11^ to 1.0 × 10^−9^ M	2.95 pM	--	--	Au nanoparticle/ toluidine blue–graphene oxide-modified electrodes were used.	[151]
**3**	EGFR T790 M mutation 16 drug-sensitive mutations	Optical	--	1–4 copies	3–5 min	Plasma	Detection of MDR leukemia Vertically aligned multi-walled carbon nanotubes based immunosensor used Cell-free circulating DNA analyzed Multiplex detection system Fluorescence-based detection	[154]
I.ii. Detection and monitoring of miR								
**4**	miR-121 miR-155 miR-205	Optical	1 fM to 1 nM (for miR- 121)	20.20 fM 15.32 fM 13.50 fM	--	HeLa MCF-7	Mo2B-based FL quenching platform Intracellular monitoring in live cell Fluorescence-based detection	[219]
**5**	miR-223 miR-122 miR-21	Optical		0.02 nM-10 nM	--	Liver cancer	Förster resonance energy transfer (FRET) based detection Multiplex quantification system for the miR Successful detection in 10% of serum samples was achieved.	[220]
**6**	MiR-21 Let-7d	Optical	--	33.93 pM	~2 h	--	Single-stranded DNA (ssDNA) used Detection instrumented by flow cytometry Fluorescence-based detection	[221]
**7**	miRNA 21 miRNA 31	Optical	50 to 200 fM (miRNA 21) 1.0 to 200 fM (miRNA 31)	0.20 fM (miRNA 21) 0.50 fM (miRNA 31)	<40 min	HeLa A549	Microfluidic paper-based system Laser-induced fluorescence used	[158]
**8**	miR-200a-3p	Optical	--	1 aM	1–2 h	MKN45 and SNU1	Single-base mismatch detection is possible too No amplification required	[222]
**9**	Survivin	Optical	--	827 pM	--	HeLa	Au nanoparticles used Cy3 and Cy5 dyes were used as donors FRET-based approach	[223]
**10**	miR-21	Optical	1 pM to 10 nM	55 fM	--	MCF7	Cyclic enzymatic amplification was achieved with help of a periodic nanostructure sensor chip Photonic biosensor	[224]
**11**	miR-34a	Electrochemical	5 to 35 μg/mL	7.52 μg/mL	~1 min	--	Label-free voltammetric detection	[225]
**12**	miR-21	Optical	5 pM to 200 nM	0.5 pM	--	Serum	Graphdiyne/graphene quantum dots were used. Detection of MCF-7 cells and live imaging of MB231 done FRET-based detection	[226]
**13**	miR-K12–5-5p	Optical	--	0.884 nM	--	Breast cancer	SERS based biosensor GaN with Au/Ag used as the platform	[227]
**14**	miR 410	Electrochemical	10 fM to 300 pM	3.90 fM	5 min	Prostate cancer cells and serum	Disposable Au nanoparticles -peptide nanotubes used Impedimetric biosensor	[159]
**15**	miR 21 miRNA-196a-5p	Optical	10 pM to 10 mM	3.31 pM 2.18 pM	30 min	Non-small cell lung cancer	SERS based biosensor Catalytic hairpin assembly-based SERS-LFA strip	[228]
**16**	miR- 141	Electrochemical	10 pM to 10 aM	3.23 aM	--	Prostrate and breast cancer	Sensing platform based on atom radical polymerization	[229]
**17**	miR-593 miR-155	Optical	5 nM to 50 nM	0.17 nM 0.25 nM	<3 h	Body fluids and tumor tissues	FRET-based biosensor Breast cancer cell biomarkers are being identified. Based on core-shell upconversion NP and MoS2 nanosheets were used.	[230]
II. PHENOTYPIC BIOSENSORS								
II.i. Phenotypic Biosensors Detecting Drug Efflux Pump								
**18**	P-glycoprotein (Pgp)	Optical	1.1 × 10^7^ − 2 × 10^3^ cells/mL	27 cells/mL	--	Chronic myeloid leukemia	Fermi level fluctuation induced charge transfer-based biosensor	[193]
**19**	Pgp	Electrochemical	50 and 100,000 cells/mL	23 ± 2 cells/mL	--	MDR_CC_	Amperometric biosensor Monoclonal Pgp antibodies and amino phenylboronic acid used as BRE immobilized over Au nanoparticles	[192]
**20**	Pgp	Optical	1.5 × 10^2^ to 1.5 × 10^7^ cells/mL	10 cells/mL	--	K562 cells	Fluorescence-based detection Detection of MDR leukemia Vertically aligned multi-walled carbon nanotubes based immunosensor used	[231]
**21**	MRP2 protein	Optical	--	1 cell	--	HepG2	Fluorescence-based detection A multifunctional gradients-customizing microfluidic device	[194]
**22**	Cell itself	Electrochemical	--	~50 cells	<12 min	Leukemia K562	Au nanoparticles -modified glassy carbon electrodes (GCE) used	[190]
II.ii. Phenotypic Biosensors Observing Cellular Profile and TME								
**23**	Membrane protein profile	Optical	--	200 cells	--	MDA-MB-231	Fluorescence-based detection. Healthy, cancerous, and metastatic human breast cancer cells can be differentiated. Dual ligand co-functionalized Au-clusters	[191]
**24**	pH	Optical	pH range of 4.2–6.4 with a pKa value of 5.18	--	~1 min	RAW 264.7	Pyrido [1,2-a] benzimidazole derivative-based fluorescent probe	[214]
**25**	Nitroreductase (NTR)	Optical	0 to 20 μg/mL	26 ng/mL	--	A549 A549/DDP	NTR is overexpressed in highly hypoxic TME Detection based on fluorescence	[213]
**26**	NTR	Optical	0 to 4 μg/mL	18.6 and 33.2 ng/mL of NTR for 1-NO2 and 2-NO2	-	A2058	Detection based on fluorescence. Sensors were designed based on the conjugation of pyridazine-1,3a,6a- triazapentalene to a para-nitrophenyl, forming two probes denoted as 1- NO2 and 2-NO2. Reduction by NTR led to the over 15- fold enhancement of fluorescence intensity in both probes.	[212]
**27**	CD59 protein	Electrochemical	1 fg/mL and 1000 fg/mL	0.38 ± 0.03 fg/mL	10 min	Saliva	Impedimetric label free biosensor Protein probe (anti CD59 antibody) immobilized over a self-assembled monolayer of Cys over Au electrode	[200]
II.iii. Phenotypic Biosensors Detecting Enzymes and Proteins								
**28**	Glutathione	Optical	0 to 80 μM	87 nM	--	HeLa HepG2 LO2	Fluorescence-based biosensor Nanoscale metal-organic frameworks are used. Two-photon imaging used	[209]
**29**	Brain-Derived Neurotrophic Factor (BDNF)	Electrochemical	4.0 to 600.0 pg/mL	1.5 ± 0.012 pg/mL	--	Serum PC12 SH-SY5Y	Microfluidics based immunosensor Biconjugate probe consisting of anti-BDNF and toluidine blue Was used to study the effects of nicotine, ethanol, and potassium ion on BDNF expression in cancer lines.	[202]
**30**	β1 Integrin	Electrochemical	1.0 × 10^4^ to 2.0 × 10^6^ cells/mL^−1^	3.5 × 10^3^ cells/ mL	--	HeLa	Impendence spectroscopy-based sensor. Glass carbon electrode impinged with mouse anti-human integrin β 1 monoclonal antibody	[198]
**31**	Intracellular telomerase	Optical	0 to 20,000 cells	280 A549 cells	--	A549 HepG2 MCF-7	Fluorescence-based biosensor. Nanoflare and hybridization chain reaction (HCR)-based signal amplification was applied together with gold/carbon nanosphere	[203]
**32**	DNA methyl transferase	Optical	0.1 to 0.2 U/μL	1.6968 × 10^−4^ U/μL	--	--	Fluorescence-based biosensor Dumbbell-shaped DNA template copper nanoparticles used	[211]
III. Biosensors Based On Drug Pharmacokinetics								
**33**	Exosomes and exosomal cisplatin	Optical	Cisplatin: 0 to 0.2 µg/mL	Cisplatin: 0.17 µg/mL Exosome: 65 nM	--	OVCA	SER based biosensor Cysteine-capped gold nanoparticles used Diagnosis of chemoresistance with accuracy greater than 90%	[216]
**34**	Methotrexate	Optical	28 to 500 nM	155 nM	--	Serum and clinical samples	Multichannel SPR-based instrument	[215]
**35**	Daunomycin Residue	Electrochemical	-- Linearity coefficient: 0.995	--	1 h	K562/A02 cells	Based on carbon nanotubes–drug interaction. Nanocomposites of daunorubicin and CNT were used.	[232]

## Data Availability

Not applicable.
